# CellProfiler 3.0: Next-generation image processing for biology

**DOI:** 10.1371/journal.pbio.2005970

**Published:** 2018-07-03

**Authors:** Claire McQuin, Allen Goodman, Vasiliy Chernyshev, Lee Kamentsky, Beth A. Cimini, Kyle W. Karhohs, Minh Doan, Liya Ding, Susanne M. Rafelski, Derek Thirstrup, Winfried Wiegraebe, Shantanu Singh, Tim Becker, Juan C. Caicedo, Anne E. Carpenter

**Affiliations:** 1 Imaging Platform, Broad Institute of Harvard and MIT, Cambridge, Massachusetts, United States of America; 2 Skolkovo Institute of Science and Technology, Skolkovo, Moscow Region, Russia; 3 Moscow Institute of Physics and Technology, Dolgoprudny, Moscow Region, Russia; 4 Allen Institute for Cell Science, Seattle, Washington, United States of America; National Cancer Institute, United States of America

## Abstract

CellProfiler has enabled the scientific research community to create flexible, modular image analysis pipelines since its release in 2005. Here, we describe CellProfiler 3.0, a new version of the software supporting both whole-volume and plane-wise analysis of three-dimensional (3D) image stacks, increasingly common in biomedical research. CellProfiler’s infrastructure is greatly improved, and we provide a protocol for cloud-based, large-scale image processing. New plugins enable running pretrained deep learning models on images. Designed by and for biologists, CellProfiler equips researchers with powerful computational tools via a well-documented user interface, empowering biologists in all fields to create quantitative, reproducible image analysis workflows.

## Author summary

The “big-data revolution” has struck biology: it is now common for robots to prepare cell samples and take thousands of microscopy images. Looking at the resulting images by eye would be extremely tedious, not to mention subjective. Thus, many biologists find they need software to analyze images easily and accurately. The third major release of our free open-source software CellProfiler is designed to help biologists working with images, whether a few or thousands. Researchers can download an online example workflow (that is, a “pipeline”) or create their own from scratch. Pipelines are easy to save, reuse, and share, helping improve scientific reproducibility. In this release, we’ve added the capability to find and measure objects in three-dimensional (3D) images. We’ve also made changes to CellProfiler’s underlying code to make it faster to run and easier to install, and we’ve added the ability to process images in the cloud and using neural networks (deep learning). We’ve also added more explanations to CellProfiler’s settings to help new users get started. We hope these changes will make CellProfiler an even better tool for current users and will provide new users better ways to get started doing quantitative image analysis.

## Introduction

### Bioimaging software ecosystem

Image analysis software is now used throughout biomedical research in order to reduce subjective bias and quantify subtle phenotypes when working with microscopy images. Automated microscopes are further transforming modern research. Experiments testing chemical compounds or genetic perturbations can reach a scale of many thousands of perturbations, and multidimensional imaging (time-lapse and three-dimensional [3D]) also produces enormous data sets that require automated analysis. In light of this data scale, computer algorithms must deliver accurate identification of cells, subcompartments, or organisms and extract necessary descriptive features (metrics) for each identified object.

Racing to keep up with the advancement of automated microscopy are several classes of biologist-focused image analysis software, such as companion packages bundled with imaging instruments (e.g., MetaMorph—Molecular Devices, Elements—Nikon), stand-alone commercial image processing tools (e.g., Imaris—Bitplane), and free open-source packages (e.g., ImageJ/Fiji, CellProfiler, Icy, KNIME). Commercial software is often convenient to use, especially when bundled with a microscope. Although cost and lack of flexibility may limit adoption, there is a focus on usability, particularly for applications of interest to the pharmaceutical industry. Still, the proprietary nature of the code in commercial software limits researchers from knowing how their data is being analyzed or modifying the strategy of a given algorithm, if desired.

The open-source biological image analysis software ecosystem is thriving [1]. ImageJ [2] was the first and is still the most widely used package for bioimage analysis; several other packages are based on its codebase (most notably, Fiji). ImageJ excels at the analysis of individual images, with a user interface analogous to Adobe Photoshop. Its major strength is its community of users and developers who contribute plugins, although an associated drawback is the sheer number of plugins, with varying degrees of functional overlap, usability, and documentation. Multitasking toolboxes like KNIME [3] offer a more modular approach, which is better suited to automated workflows. KNIME equips users with a wide breadth of powerful utility, from performing image analysis to data analytics.

### CellProfiler

CellProfiler, our open-source software for measuring and analyzing cell images, has been cited more than 6,000 times, currently at a rate of more than 1,000 per year. The first version of CellProfiler was introduced in 2005 and published in 2006 [4]. It is widely adopted worldwide, enabling biologists without training in computer vision or programming to quantitatively measure phenotypes robustly from thousands of images. A second major version of CellProfiler, rewritten in Python from its original MATLAB implementation, was published in 2011 [5] and included methods for tracking cells in movies and measuring neurons, worms, and tissue samples. In 2015, a laboratory unaffiliated with our team rigorously compared 15 free software tools for biological image analysis: CellProfiler was ranked first for both usability and functionality [6].

CellProfiler provides advanced algorithms for image analysis, organized as individual modules that can be placed in sequential order to form a pipeline. This pipeline is then used to identify and measure cells or other biological objects and their morphological features. CellProfiler’s modular design and carefully curated library of image processing and analysis modules benefits biologists in several ways:

Reproducibility at scale: CellProfiler is designed to produce high-content information for each cell or other object of interest in each image and to apply the same objective analysis in high-throughput, e.g., across thousands or millions of images.

Flexible feature extraction: Individual modules measure standard morphological features such as size, shape, intensity, and texture. Customized combinations of modules can extract even more complex information. As such, CellProfiler is commonly used for morphological profiling experiments such as Cell Painting [7,8], which is being adopted in pharmaceutical companies to speed several steps in drug discovery [9].

Easy to learn: Each of the 70+ modules includes carefully crafted documentation, curated by both imaging and biology experts, to make image processing more approachable and understandable for the average scientist. Further, each individual setting is explained in practical terms to aid researchers in configuring it. The number of modules and settings is carefully limited to avoid overwhelming users, while a plugin system allows the flexibility of a larger array of contributed modules.

Community: CellProfiler has an active community of more than 3,000 people on its online question and answer forum. With more than 15,000 posts, users provide feedback that fuels improvements to CellProfiler, find pipelines related to their area of research, interact with developers, get input on challenging problems, and improve image analysis skills and knowledge by helping other users design solutions.

## Results

In the CellProfiler 3.0 release, we introduced methods for analyzing 3D images, using deep learning architectures and cloud computing resources, and other improvements to CellProfiler’s usability and capabilities.

### High-throughput 3D analysis

This new version of CellProfiler has support for analysis of 3D images in many of its modules (S1 Fig). Although open-source software tuned to 3D problems exists (e.g., Vaa3D, BioImageXD, Slicer) [10], it often emphasizes visualization and rendering; these new 3D capabilities of CellProfiler meet the community’s demand for modular high-throughput 3D analysis. CellProfiler 3.0 can apply image processing, segmentation, and feature extraction algorithms to entire image volumes (volumetric analysis), in addition to the more typical iterative and separate analysis of two-dimensional slices from a 3D volume (“plane-wise” analysis). Whole-volume algorithms consider 3D neighborhoods and incorporate information from surrounding planes, yielding more accurate results, but require more available memory, particularly for large files. CellProfiler’s volumetric algorithms can be configured to account for anisotropic data (in which the distance between Z planes does not match the distance between pixels in the X and Y dimensions). While we focused on adding 3D capability to most of our image processing and feature extraction modules, we will continue increasing the number of CellProfiler modules that support image volumes for situations in which it is not computationally prohibitive.

**Fig 1:**
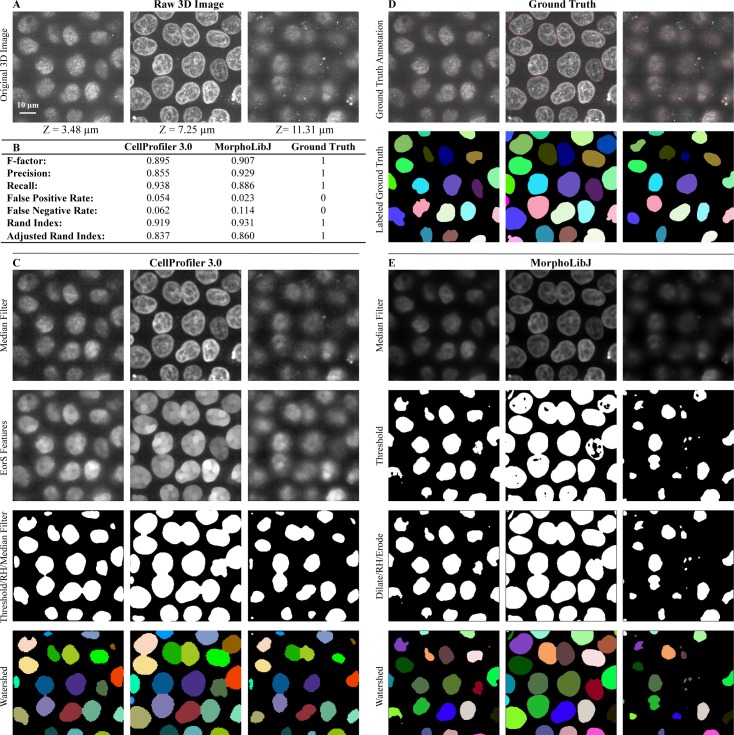
Volumetric processing for 3D images of DNA-stained nuclei of hiPSCs using CellProfiler 3.0. Images are from the Allen Institute for Cell Science, Seattle, and available from the Broad Bioimage Benchmark Collection (https://data.broadinstitute.org/bbbc/BBBC034/). (A) Original 3D image of nuclei monolayer prior to analysis. (B) Evaluation of CellProfiler 3.0 performance in comparison to the MorphoLibJ plugin in Fiji software. Both were compared to manually annotated ground truth using CellProfiler’s MeasureImageOverlap module. (C) Selected CellProfiler 3.0 image processing modules used for hiPSC nucleus segmentation. Figure labels: RH (“RemoveHoles”), EorS Features (“EnhanceOrSuppressFeatures”). (D) Ground truth obtained by manual annotation of each Z-slice using GIMP software. (E) Image processing done using Fiji’s MorphoLibJ plugin (macro code is presented in S1 Table). 3D, three-dimensional; hiPSC, human induced pluripotent stem cell.

**Fig 2:**
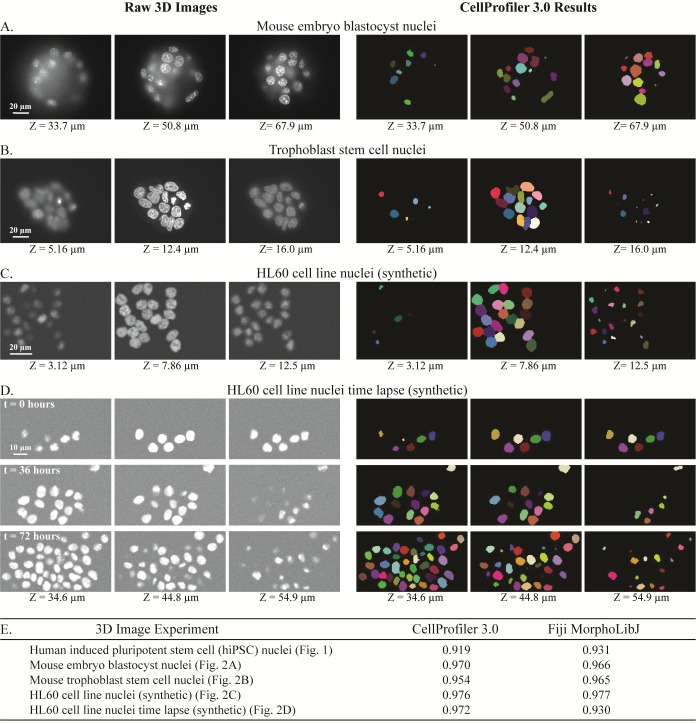
Examples of 3D image segmentation produced by CellProfiler 3.0, across two experimental systems and two sets of synthesized images. Three focal planes shown for each. Raw images (left) and CellProfiler outputs (right) showing nuclei of mouse embryo blastocyst (A), mouse trophoblast stem cells (B), and synthetic images of HL60 cell lines (C) and (D). More information about segmentation steps used for these images can be found in S2–S5 Figs. (E) Comparison of the segmentation accuracy of CellProfiler 3.0 and Fiji’s plugin MorphoLibJ, based on the Rand index of the processed image and its ground truth (out of a total of 1.0). Object accuracy comparisons of these same images may be found in S6 Fig and S3 File. 3D, three-dimensional; hiPSC, human induced pluripotent stem cell.

We developed 3D pipelines to identify cells and subcompartments of cells for a number of experimental situations and sample types across a number of laboratories. We identified nuclei based on a DNA stain (Fig 1A) in 3D image stacks of human induced pluripotent stem cells (hiPSCs). After processing by several CellProfiler modules (Fig 1C), the final results agree well with manually annotated nuclei (Fig 1D). Results for a variety of images with a range of complexity are shown in Fig 2, with more detailed views in S2–S5 Figs. We characterized CellProfiler’s segmentation accuracy in two ways: in the first, we used real microscopy images (Fig 1A, Fig 2A, Fig 2B) whose ground truth was manually annotated by an expert image analyst; such images are realistic, but the manual annotation introduces some subjectivity. We therefore also used synthetic images (Fig 2C, Fig 2D)[11,12], which, depending on the model used to create them, may not perfectly represent real microscopy images but whose ground truth can be unambiguously known.

**Fig 3:**
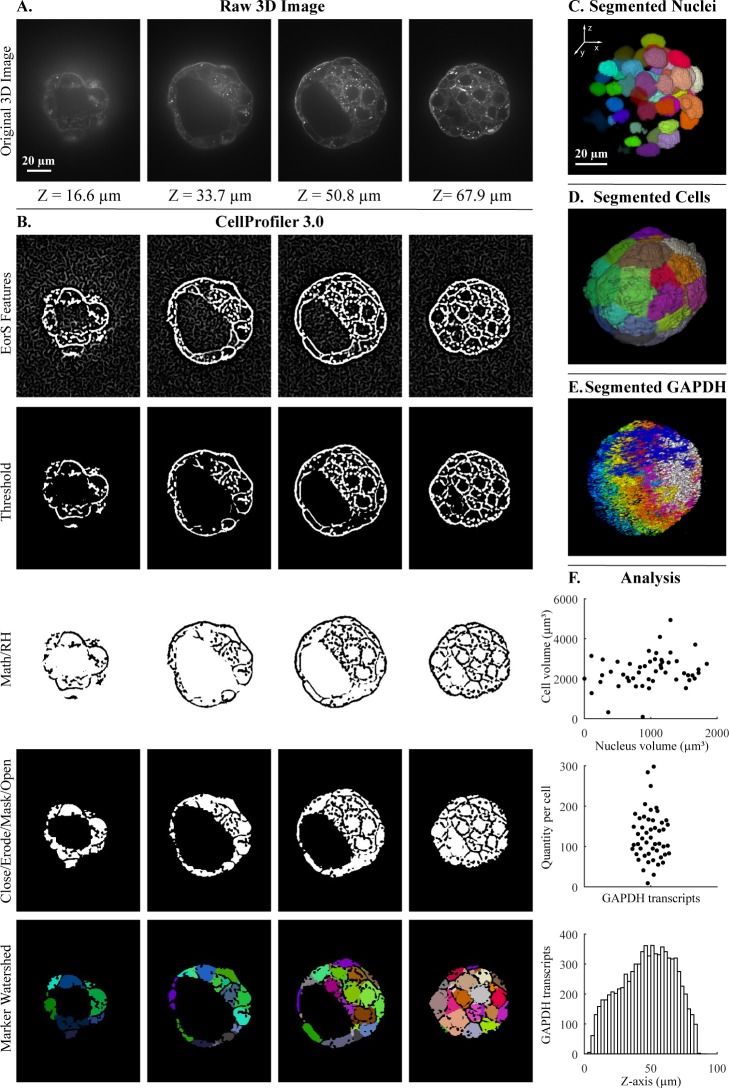
Segmentation steps for the quantification of transcripts per cell within a 3D blastocyst. Images were captured of a mouse embryo blastocyst cell membrane stained with WGA and FISH for GAPDH transcripts. (A) Original 3D image of blastocyst cell membrane prior to analysis. (B) CellProfiler 3.0 image processing modules used for membrane image processing. Figure labels: RH (“RemoveHoles”), Close (“Closing”), Erode (“Erosion”), Mask (“MaskImage”), Math (“ImageMath”), EorS Features (“EnhanceOrSuppressFeatures”). (C) Nuclei after segmentation by CellProfiler, as viewed in Fiji. (D) Segmentation of cells after setting nuclei as seeds by CellProfiler, as viewed in Fiji. (E) Segmentation of GAPDH transcript foci using CellProfiler, as viewed in Fiji. (F) Examples of analysis that can be done by CellProfiler: (top) cell volume relative nucleus volume, (middle) GAPDH transcript quantity in each cell using CellProfiler’s “RelateObjects” module, (bottom) number of GAPDH transcripts in Z-plane (bin size = 2.5 μm). The underlying measurements may be downloaded as S1 File. *Images were provided by Javier Frias Aldeguer and Nicolas Rivron from Hubrecht Institute*, *Netherlands*, *and are available from the Broad Bioimage Benchmark Collection (https://data.broadinstitute.org/bbbc/BBBC032/*). 3D, three-dimensional; FISH, fluorescent in situ hybridization; GAPDH, glyceraldehyde 3-phosphate dehydrogenase; WGA, wheat germ agglutinin.

**Fig 4:**
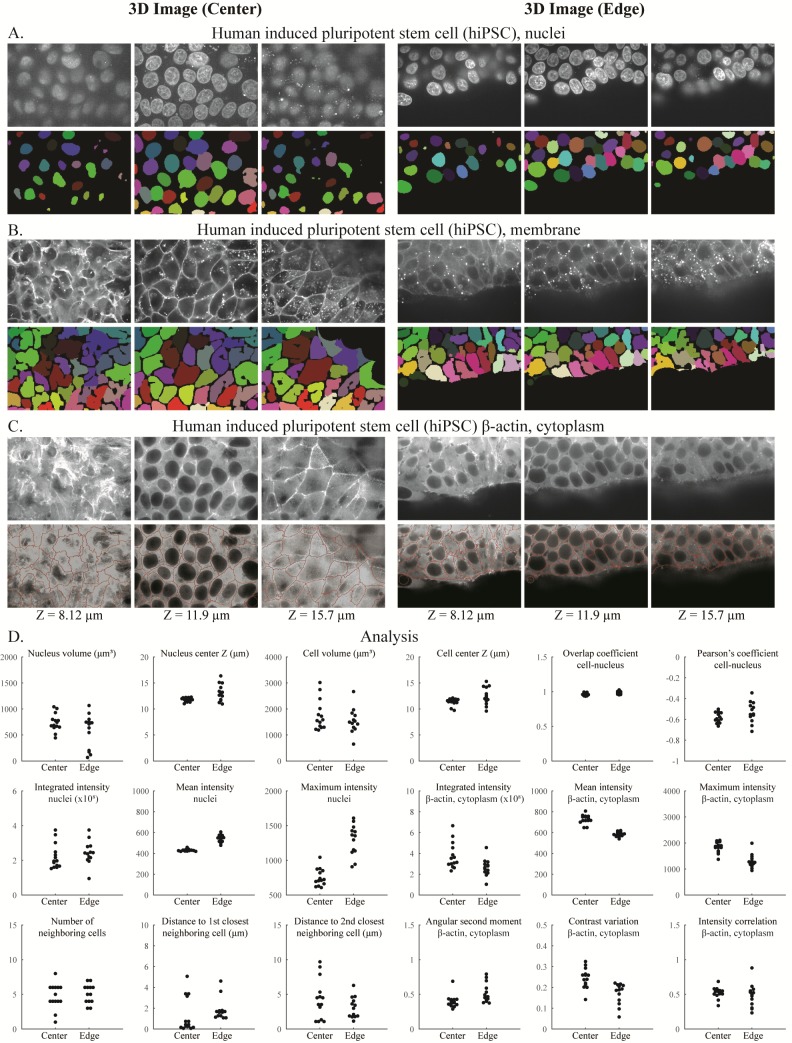
Segmentation and analysis of 3D hiPSC images using CellProfiler 3.0. DNA channel showing nuclei (A), CellMaskDeepRed channel showing membrane (B), and GFP channel showing beta-actin (C) at the center (left) and edge (right) of the hiPSC colony. (D) Various measurements obtained from the samples are shown; note that cells touching the edge of each image are excluded from this analysis. The underlying measurements may be downloaded as S2 File. *Images are from the Allen Institute for Cell Science*, *Seattle*, *and are available from the Broad Bioimage Benchmark Collection (https://data.broadinstitute.org/bbbc/BBBC034/)*. 3D, three-dimensional; GFP, green fluorescent protein; hiPSC, human induced pluripotent stem cell.

To determine how well the segmented objects agreed with ground truth, CellProfiler’s “MeasureImageOverlap” module was used to calculate the plane-wise Rand index [13], a performance metric of accuracy (Fig 1B, Fig 2E). Rand index values showed good agreement (0.919–0.976) between each tested image and its ground truth. The results produced by CellProfiler 3.0 were comparable to results produced by the commonly used Fiji plugin MorphoLibJ (0.930–0.977) (Fig 1B, Fig 2E and S2–S5 Figs; the MorphoLibJ macro codes are provided in S1 Table). We demonstrate several kinds of analysis, including analyses of cell count in a time series that was synthetically generated [11,14](S5 Fig); identification and quantification of children objects inside parent objects, such as speckles of transcripts within cells (Fig 3); and measurement of various features of hiPSCs located at the center and the edge of the cell colony (Fig 4).

All pipelines, annotated with notes to understand the function of each module, are provided at https://github.com/carpenterlab/2018_mcquin_PLOSBio. All raw images, together with ground truth annotations used to test CellProfiler 3.0 performance, are publicly available for further community algorithm development in the Broad Bioimage Benchmark Collection [15], as indicated in the legends for Fig 1 and S2–S5 Figs.

### Support for deep learning

Convolutional neural networks (CNNs) are a type of deep learning model that transforms input images into outputs specified by the problem type [16]. For instance, image classification models transform images into categorical labels [17], while image segmentation models transform images into segmentation masks [18]. CNNs are now widely used to solve many computer vision tasks, given their ability to produce accurate outputs after learning from examples. CellProfiler now can be configured to make use of cutting-edge CNNs to analyze biomedical images. While CellProfiler does not yet incorporate user-friendly functionalities to train neural networks, various models that have been already trained by researchers can be run inside CellProfiler.

Running neural network models requires the installation of certain deep learning frameworks that are distributed separately, such as TensorFlow or Caffe. TensorFlow [19] is an open-source software library for machine learning that interfaces with Python and is compatible with CellProfiler when installed from source on Linux, Mac, and more recently, Windows. Caffe [20] is a deep learning framework designed for high-performance neural networks and is primarily available for Linux systems. Some network models may need special graphics processing units (GPUs) installed and configured in the system to run the computations efficiently, but this is not always required. Fortunately, both TensorFlow and Caffe can easily switch between running on GPUs and traditional central processing units (CPUs) just by changing the corresponding configuration.

We created the CellProfiler 3.0 module ClassifyPixels-Unet to segment nuclei in images stained with DNA labels (https://github.com/CellProfiler/CellProfiler-plugins). This plugin implements a U-Net[18] model using TensorFlow and can be run on CPUs. We have also provided the network architecture with training routines in case users have their own annotated images to learn a segmentation model for different images and objects of interest (https://github.com/carpenterlab/unet4nuclei). The ClassifyPixels-Unet module classifies pixels into one of three classes: background, nucleus interior, or nuclear boundary (S7 Fig). A pretrained network for nuclei segmentation is available for download and is automatically loaded by the plugin; a pipeline and image to run this are available as S4 File.

We also created a CellProfiler 3.0 module, MeasureImageFocus, in collaboration with Google Accelerated Science, who trained a model to detect focus in images [21]. The module displays a table with the predicted focus score and certainty for the whole image, as well as a figure with the focus scores and corresponding certainties of individual 84 × 84 patches represented by color and opaqueness. It uses TensorFlow as its underlying deep learning framework. Independently, Sadanandan and colleagues created a CellProfiler 2.2.0 module—CellProfiler-Caffe bridge—that enables running a pretrained model for cell segmentation within a CellProfiler pipeline [22].

### Cloud computing

We created Distributed-CellProfiler (https://github.com/CellProfiler/Distributed-CellProfiler), a script-based interface that allows running thousands of batches of images through CellProfiler in parallel on Amazon Web Services (AWS; S8 Fig). While Distributed-CellProfiler does require basic knowledge of AWS and interaction with the command line, it is well documented and has been successfully run by biologists without formal computational training. The script handles infrastructure creation and removal as well as creation and storage of logs, allowing users without access to a local cluster computing environment to analyze large data sets with only minimal time devoted to having to set up those resources. Sample pipelines and configuration files are available as S5 File.

### Improvements for CellProfiler 3.0

Plug-ins: CellProfiler-plugins is a new repository for the community to share and distribute new CellProfiler modules (https://github.com/CellProfiler/CellProfiler-plugins).

Documentation: All of CellProfiler’s documentation was updated for content and readability; detailed help is available for 100% of module configuration options (excluding plugins).

New image processing features: CellProfiler 3.0 introduces an extended suite of modules for feature detection, feature extraction, filtering and noise reduction, image processing, image segmentation, and mathematical morphology operations.

Infrastructure improvements: The project team reengineered major core components of CellProfiler. CellProfiler’s codebase was trimmed down, in part because of better integration with Python’s scientific community. We have adopted and contributed to the standard libraries of the scientific Python community, including NumPy, SciPy, and scikit-image. CellProfiler’s code is now 100% Python, which improves interoperability with the robust Python scientific ecosystem and simplifies third-party contributions. As well, we upgraded support to 64-bit on Linux, MacOS, and Windows, and a continuous integration process ensures the software is well tested on a variety of platforms.

We made substantial progress simplifying CellProfiler’s installation. In addition to our previously existing Mac and Windows builds, a Python wheel is now available from the Python Package Index, and a Docker image is now available from Docker Hub. In an effort to expand CellProfiler’s flexibility, we made CellProfiler much simpler to compile on a variety of familiar and unusual platforms by requiring fewer dependencies and only using ubiquitous build systems.

Educational resources: CellProfiler’s many examples and tutorials are now publicly available on GitHub (https://github.com/CellProfiler/examples and https://github.com/CellProfiler/tutorials) and have been updated for compatibility with CellProfiler 3.0.

Speed: CellProfiler 3.0’s processing speed is faster than version 2.2 on the most common types of pipelines; the degree of difference depends on the exact modules involved: CellProfiler 3.0 ran at a comparable or faster speed than CellProfiler 2.2 for 11 of 16 example pipelines tested (S9 Fig). While the total amount of time needed to run the five pipelines shown in S9 Fig was comparable between CellProfiler and MorphoLibJ (482 versus 542 seconds), the relative speed was highly specific to the individual pipeline (S6 File), ranging from 2× faster in CellProfiler to 6× faster in MorphoLibJ (S2 Table). In addition, CellProfiler can run multiple images in parallel, depending on the individual’s number of threads, computing power, and access to cloud computing resources, making it suited to large-scale experiments. As well, CellProfiler’s modules enable more readily configurable complex analyses than MorphoLibJ, such as associating cytoplasm regions (as in Fig 3), transcripts (as in Fig 3), and other entities to nuclei and measuring a wide variety of morphological properties of each, including intensities, shapes, textures, colocalization metrics, and neighborhood relationships (as in Fig 4).

## Future directions

CellProfiler is mature software serving a large community and making an impact through its thousands of users’ biological discoveries. It has been involved in the discovery of potential life-saving drugs for infectious diseases, leukemia, and cerebral cavernous malformation [23–27] and in clinical trials for hematological malignancies [28] and will continue to fuel basic and applied research around the world.

CellProfiler can readily generate a large amount of morphological information for each biological entity that is measured. We see advancements in data mining, downstream and apart from CellProfiler, as blossoming in the coming years. Already, 20 laboratories in the field of morphological profiling have gathered for two annual meetings/hackathons (now called CytoData) [29], collaborated to outline best practices [30], and begun a community library (Cytominer, https://github.com/cytomining/cytominer). In addition to our user-friendly tool for classical machine learning based on measured features, CellProfiler Analyst [31], we have begun creating Deepometry (http://github.com/broadinstitute/deepometry), a tool that enables scientists without training in machine learning to perform single-cell phenotype classification using deep learning and other advanced downstream data analytics. Interoperability of CellProfiler with popular notebook tools like Jupyter would allow seamless workflows involving other complementary software tools.

Finally, deep learning has revolutionized computer vision and other fields in the past few years [16,32], and bioimaging will be no exception. As noted, already some models trained for specific tasks can be used via CellProfiler, and we expect that over time, more generalizable models will be created that can accomplish useful tasks such as detecting common cellular structures across diverse types of images and experimental setups, as in, for example, the 2018 Data Science Bowl challenge. Community-driven collections of images and ground truth, as well as “model zoos,” will be instrumental for this. We have also begun creating libraries (Keras-ResNet [https://github.com/broadinstitute/keras-resnet] and Keras-RCNN [https://github.com/broadinstitute/keras-rcnn]) that will provide the foundation for interfaces that allow biologists to annotate, train, and use deep learning models. We expect that over time, these models will reduce the amount of time biologists spend tuning classical image processing algorithms to identify biological entities of interest in images.

## Materials and methods

### Blastocyst and trophoblast cell imaging

Images were kindly provided by Javier Frias Aldeguer and Nicolas Rivron of Hubrecht Institute for Developmental Biology and Stem Cell Research and Li Linfeng of MERLN Institute for Technology-Inspired Regenerative Medicine. As per Rivron and colleagues [33], mouse embryos (3.5 dpc) were fixed right after isolation from the mother’s uterus. Fixation was performed using 4% PFA in RNAse-free PBS containing 1% acetic acid. ViewRNA ISH Cell Assay kit (cat# QVC0001) was used for performing smFISH on the embryos. The protocol includes steps of permeabilization and protease treatment as well as probes, preamplifier, amplifier, and label hybridizations. Embryos were then mounted in Slowfade reagent (Thermofisher cat# S36937) and directly imaged in a PerkinElmer Ultraview VoX spinning disk microscope in confocal mode by using a 63×/1.40 NA oil immersion lens.

### hiPSC culture, staining and imaging

Images were acquired by collaborators from the Allen Institute for Cell Science, Seattle, as per Roberts and colleagues [34]. Briefly, wild-type C (WTC) hiPSCs were cultured in a feeder-free system on tissue culture dishes or plates coated with GFR Matrigel (Corning) diluted 1:30 in cold DMEM/F12 (Gibco). Undifferentiated cells were maintained with phenol red containing mTeSR1 media (85850, STEMCELL Technologies) supplemented with 1% (v/v) penicillin-streptomycin (P/S; Gibco). Cells were not allowed to reach confluency greater than 85% and are passaged every 3–4 days by dissociation into single-cell suspension using StemPro Accutase (Gibco). When in single-cell suspension, cells were counted using a Vi-CELL Series Cell Viability Analyzer (Beckman Coulter). After passaging, cells were replated in mTeSR1 supplemented with 1% P/S and 10 μM ROCK inhibitor (Stemolecule Y-27632, Stemgent) for 24 hours. Media is replenished with fresh mTeSR1 media supplemented with 1% P/S daily. Cells were maintained at 37°C and 5% CO2. Cells were maintained with phenol red–free mTeSR1 media (05876, STEMCELL Technologies) 1 day prior to live cell imaging.

Three to four days after cells are plated and mature and healthy colonies are observed on 96- and 24-well imaging plates, the cells are stained with NucBlue Live ready probe reagent (R37605, ThermoFisher) and CellMask Deep Red plasma membrane stain (C10046, ThermoFisher) to visualize DNA and plasma membrane, respectively. The protocol is available online: http://www.allencell.org/uploads/8/1/9/9/81996008/sop_for_cellmask-and-nucblue_v1.0_1.pdf. Phenol red–free mTeSR1 is preequilibrated to 37°C and 5% CO2. 1X NucBlue solution made in preequilibrated phenol red–free mTeSR1 is spun for 60 minutes at 20,000 g. The 2X and 10X working stocks of CellMask Deep Red lot #1730970 and #1813792, respectively, are made in 1X NucBlue solution. All solutions are kept at 37°C and 5% CO2 until used. The 100 μL and 400 μL of NucBlue solution are added per well of 96-well imaging plates and 24-well imaging plates, respectively, and incubated at 37°C and 5% CO2 for 20 minutes. An equal amount of CellMask Deep Red working stock is added to the wells containing NucBlue solution. Final dye concentrations in the wells are 1X NucBlue and 1X and 5X CellMask Deep Red lots #1730970 and #1813792, respectively. Cells are incubated at 37°C and 5% CO2 for 10 minutes and gently washed with preequilibrated phenol red–free mTeSR1. Fields of view as shown in Fig 4 that are acquired near the edge (and the center as a control) of hiPSC colonies receive an additional photoprotective cocktail treatment which serves to minimize singlet oxygen and free radical formation. The photoprotective cocktail is used at a working concentration of 0.3 U/ml (1:100) OxyFluor as defined by the OxyFluor product insert, with the addition of 10 mM sodium lactate and 1 mM ascorbic acid (OxyFluor OF-0005, Oxyrase).

As per Roberts and colleagues [34], cells were imaged on a Carl Zeiss spinning disk microscope with a Carl Zeiss 20×/0.8 NA plan APOCHROMAT or 100×/1.25 W C-APOCHROMAT Korr UV Vis IR objective, a CSU-X1 Yokogawa spinning disk head, and Hamamatsu Orca Flash 4.0 camera. Microscopes were outfitted with a humidified environmental chamber to maintain cells at 37°C with 5% CO2 during imaging. Cells are imaged immediately following the wash step and for up to 2.5 hours after dye addition on a Zeiss spinning disk microscope at 100× with the following general settings: 405 nm at 0.28 mW, 200 ms exposure; 638 nm at 2.4 mW, 200 ms exposure; acquiring each channel at each z-step.

### Generation of ground truth annotations

Experienced bioimage analysts drew outlines around nuclear boundaries on each slice of the 3D images and labeled background regions in a different color with GIMP (https://www.gimp.org), an open-source drawing and annotation software. These annotated layers were then exported from GIMP as an image. This outline image is converted to 3D objects via a CellProfiler pipeline (https://github.com/CellProfiler/tutorials/tree/master/Annotation), and an object label matrix image is exported, in which each object’s voxels are assigned a unique integer value. These label images are referenced as ground truth.

## Supporting information

S1 Fig Modules with support for 3D in CellProfiler. Overview of modules available in CellProfiler 3.1.0 for 3D image analysis. 3D, three-dimensional.
(TIF)

S2 Fig Segmentation steps for the analysis of mouse embryo blastocyst nuclei stained with Hoechst. Images are available from the Broad Bioimage Benchmark Collection (https://data.broadinstitute.org/bbbc/BBBC032/), as in Fig 2A of the main paper. (A) Original 3D image of blastocyst nuclei prior to analysis. (B) Evaluation of CellProfiler 3.0 performance in comparison to the MorphoLibJ plugin in Fiji software. Both were compared to manually annotated ground truth using CellProfiler’s MeasureImageOverlap module. (C) CellProfiler 3.0 image processing modules used for blastocyst nuclei segmentation. (D) Ground truth obtained by manual annotation of each Z-slice using GIMP software. (E) Image processing done using Fiji’s MorphoLibJ plugin (macro code is presented in S1 Table). *Images were obtained using PerkinElmer Ultraview VoX spinning disk microscope with a 63× immersion objective (distance between Z-slices = 0*.*5* μ*m) and provided by Javier Frias Aldeguer and Nicolas Rivron from Hubrecht Institute*, *Netherlands*. 3D, three-dimensional.
(JPG)

S3 Fig Segmentation steps for the analysis of mouse trophoblast stem cell nuclei stained with Hoechst. Images are available from the Broad Bioimage Benchmark Collection (https://data.broadinstitute.org/bbbc/BBBC033/), as in Fig 2B of the main paper. (A) Original 3D stem cell nuclei image prior to analysis. (B) Evaluation of CellProfiler 3.0 performance in comparison to the MorphoLibJ plugin in Fiji software. Both were compared to manually annotated ground truth using CellProfiler’s MeasureImageOverlap module. (C) CellProfiler 3.0 image processing modules used for stem cell nuclei. (D) Ground truth obtained by manual annotation of each Z-slice using GIMP software. (E) Image processing done using Fiji’s MorphoLibJ plugin (macro code is presented in S1 Table). *Images were obtained using a PerkinElmer Ultraview VoX spinning disk microscope with a 63× oil immersion objective (distance between Z-slices = 0*.*5* μ*m) and provided by Javier Frias Aldeguer and Nicolas Rivron from Hubrecht Institute*, *Netherlands*, *and ground truth was annotated by Li Linfeng from MERLN Institute*, *Netherlands*. 3D, three-dimensional.
(JPG)

S4 Fig Segmentation steps for the analysis of synthetic images depicting HL60 cell line nuclei. Images are available from the Broad Bioimage Benchmark Collection (https://data.broadinstitute.org/bbbc/BBBC024/), as in Fig 2C of the main paper. Synthetic images with 75% clustering probability and low SNR were chosen for analysis. The data set was generated using CytoPacq [12] set up to simulate a Zeiss Axiovert S100 microscope (objective Zeiss 63×/1.40 Oil DIC) attached to confocal unit Atto CARV and CCD camera Micromax 1300-YHS. (A) Original 3D image of HL60 nuclei prior to analysis. (B) Evaluation of CellProfiler 3.0 performance in comparison to the MorphoLibJ plugin in Fiji software. Both were compared to manually annotated ground truth using CellProfiler’s MeasureImageOverlap module. (C) CellProfiler 3.0 image processing modules used for HL60 cell nucleus segmentation. (D) Computer-generated ground truth. (E) Image processing done using Fiji’s MorphoLibJ plugin (macro code is presented in S1 Table). 3D, three-dimensional; CCD, charge-coupled device; SNR, signal-to-noise ratio.
(JPG)

S5 Fig Segmentation steps for the analysis of synthetic images from the cell tracking challenge (http://www.celltrackingchallenge.net) depicting HL60 cell line nuclei. Images are taken from the Broad Bioimage Benchmark Collection (https://data.broadinstitute.org/bbbc/BBBC035/), as in Fig 2D of the main paper. (A) Original 3D image of HL60 nuclei prior to analysis. (B) CellProfiler 3.0 image processing modules used for HL60 cell nucleus segmentation. (C) Watershed obtained using Fiji’s MorphoLibJ plugin (macro code is presented in S1 Table). (D) Computer-generated ground truth. (E) Number of identified nuclei in six 3D images representing six different time points. (F) Evaluation of CellProfiler 3.0 performance (average and standard deviation of six images) in comparison to Fiji’s MorphoLibJ plugin. Both were compared to manually annotated ground truth using CellProfiler’s MeasureImageOverlap module. *The data set was created by Vladimir Ulman and David Svoboda (Masaryk University*, *Czech Republic) using MitoGen*, *part of CytoPacq* *[12]*, *to model a Zeiss Axiovert S100 microscope attached to confocal unit Atto CARV with a Micromax 1300-YHS camera with a Plan-Apochromat 40×/1*.*3 (oil) objective lens* *[11,14]*. 3D, three-dimensional.
(JPG)

S6 Fig Accuracy of nuclear segmentation using CellProfiler and MorphoLibJ. The fraction of nuclei correctly identified relative to their ground truth was assessed for both CellProfiler (solid line) and MorphoLibJ (dashed line) for the results shown in Fig 1 and S2–S5 Figs. A nucleus was considered correctly segmented at a given threshold if the intersection of the voxels of the ground truth and segmented nuclear volumes was greater than the threshold times the union of the voxels; small errors in segmentation are tolerated at lower thresholds but not at higher thresholds. CellProfiler met or exceeded the fraction correctly identified for most thresholds for 4 of 5 test images. Images and code needed to reproduce these results are available as S3 File.
(PNG)

S7 Fig Segmentation of U2OS cells in images, using the deep learning based ClassifyPixels-Unet plugin. Image is available from the Broad Bioimage Benchmark Collection (https://data.broadinstitute.org/bbbc/BBBC022/, filename XMtest_B12_s2_w19F7E0279-D087-4B5E-9899-61971C29CB78.tif, see S4 File). The U-Net model was trained using 150 manually annotated DAPI images from the same collection. Implementation and training framework is available at https://github.com/carpenterlab/unet4nuclei. (A) The prediction for the three classes (background, boundary, and nuclei) is calculated for each image. (B) Raw image and nuclei segmentation using ClassifyPixels-Unet and IdentifyPrimaryObjects modules, with objects touching the edge excluded. Pipeline and image available as S4 File.
(PNG)

S8 Fig Distributed-CellProfiler enables processing thousands of images in parallel. A data set of seventeen 384-well plates was processed using Distributed-CellProfiler on an AWS cluster. Each plate comprised 3,456 five-channel images (2,160 × 2,160 pixels). A CellProfiler pipeline was run on each image to identify cells and then extract measurements per cell. In all, 12,415,665 cells were identified, and 2,191 measurements were made per cell. It would have taken more than five months to analyze the data set using a single machine with 16 vCPUs. Using a cluster of 195 such machines on AWS, this data set was processed in less than 21 hours and cost $765 in total. The graph shows the number of pending images over the 21-hour period of processing this data set. The configuration files used to process this data set are provided in S5 File). AWS, Amazon Web Services; vCPU, virtual central processing unit.
(PNG)

S9 Fig Comparison of runtimes between CellProfiler 3.0 and CellProfiler 2.2. (Toward the left: CellProfiler 3.0 is faster; toward the right: CellProfiler 2.2 is faster). We compared the performance of example pipelines (available at https://github.com/CellProfiler/examples and http://cellprofiler.org) for CellProfiler 2.2 and 3.0 on OS X 10.12.6 (2.8 GHz Intel Core i7 and 16 GB 1600 MHz DDR3.). The figure above shows the difference of mean runtimes between CellProfiler 3.0 and 2.2 across 10 identical image sets. CellProfiler 3.0 demonstrates improved or comparable performance to CellProfiler 2.2 in 11 of 16 example pipelines, and these represent the more commonly used applications.
(PNG)

S1 Table Fiji macros used for each file. Macro code constructed in the MorphoLibJ plugin for Fiji software that was used for analysis of 3D image examples presented in Fig 1 and S2–S5 Figs.
(XLSX)

S2 Table Image segmentation speed comparison of CellProfiler and the MorphoLibJ 1.3.3 plugin in Fiji (ImageJ 1.51n) on x-64 PC (Dell 7280) Windows 10 Pro (2.8 GHz Intel Core i7-7600U CPU and 16 GB 2133 MHz DDR4 SDRAM). Units of time are in seconds. One image representing the 72-hour time point (72 hours) was used to compare CellProfiler 3.0 and Fiji presented in S5 Fig.
(XLSX)

S1 File CellProfiler measurements of mouse blastocysts. Measurements of mouse blastocyst cells and GAPDH transcript foci created by CellProfiler; these serve as the underlying data for Fig 3F. GAPDH, glyceraldehyde 3-phosphate dehydrogenase.
(XLSX)

S2 File CellProfiler measurements of hiPSCs. Per cell measurements of hiPSCs created by CellProfiler; these serve as the underlying data for Fig 4D. hiPSC, human induced pluripotent stem cell.
(XLSX)

S3 File Code to asses per-object segmentation accuracy in CellProfiler and MorphoLibJ. A file to reproduce the results presented in S6 Fig. This contains the ground truth images, CellProfiler-produced segmentations, and MorphoLibJ-produced segmentations, as well as a Jupyter notebook that can be run from the directory once unzipped to replicate the code.
(ZIP)

S4 File Example pipeline and nuclei image for deep learning in CellProfiler 3.0. Example pipeline and image needed to run the ClassifyPixels-Unet module.
(ZIP)

S5 File Example pipelines and configuration files needed to run CellProfiler on AWS. These pipelines and files can be used to run Distributed-CellProfiler; they are specifically configured to run the Cell Painting assay [7]. AWS, Amazon Web Services.
(ZIP)

S6 File CellProfiler 3D module run times. CPU and wall-clock runtimes for individual CellProfiler modules in the example pipelines; the summary of this data can be seen in S2 Table. 3D, three-dimensional; CPU, central processing unit
(XLSX)

## Abbreviations

3D: three-dimensional
AWS: Amazon Web Services
CNN: convolutional neural network
CPU: central processing unit
FISH: fluorescent in situ hybridization
GAPDH: glyceraldehyde 3-phosphate dehydrogenase
GPU: graphics processing unit
hiPSC: human induced pluripotent stem cell
P/S: penicillin-streptomycin
WGA: wheat germ agglutin
WTC: wild-type C

## References

[1] Eliceiri KW, Berthold MR, Goldberg IG, Ibáñez L, Manjunath BS, Martone ME, et al Biological imaging software tools. Nat Methods. 2012;9: 697–710. doi: 10.1038/nmeth.2084 22743775 10.1038/nmeth.2084 PMC3659807

[2] Rueden CT, Schindelin J, Hiner MC, DeZonia BE, Walter AE, Arena ET, et al ImageJ2: ImageJ for the next generation of scientific image data. BMC Bioinformatics. 2017;18: 529 doi: 10.1186/s12859-017-1934-z 29187165 10.1186/s12859-017-1934-z PMC5708080

[3] Fillbrunn A, Dietz C, Pfeuffer J, Rahn R, Landrum GA, Berthold MR. KNIME for reproducible cross-domain analysis of life science data. J Biotechnol. 2017;261: 149–156. doi: 10.1016/j.jbiotec.2017.07.028 28757290 10.1016/j.jbiotec.2017.07.028

[4] Carpenter AE, Jones TR, Lamprecht MR, Clarke C, Kang IH, Friman O, et al CellProfiler: image analysis software for identifying and quantifying cell phenotypes. Genome Biol. 2006;7: R100 doi: 10.1186/gb-2006-7-10-r100 17076895 10.1186/gb-2006-7-10-r100 PMC1794559

[5] Kamentsky L, Jones TR, Fraser A, Bray M-A, Logan DJ, Madden KL, et al Improved structure, function and compatibility for CellProfiler: modular high-throughput image analysis software. Bioinformatics. 2011;27: 1179–1180. doi: 10.1093/bioinformatics/btr095 21349861 10.1093/bioinformatics/btr095 PMC3072555

[6] Wiesmann V, Franz D, Held C, Munzenmayer C, Palmisano R, Wittenberg T. Review of free software tools for image analysis of fluorescence cell micrographs. J Microsc. 2015;257: 39–53. doi: 10.1111/jmi.12184 25359577 10.1111/jmi.12184

[7] Bray M-A, Singh S, Han H, Davis CT, Borgeson B, Hartland C, et al Cell Painting, a high-content image-based assay for morphological profiling using multiplexed fluorescent dyes. Nat Protoc. 2016;11: 1757–1774. doi: 10.1038/nprot.2016.105 27560178 10.1038/nprot.2016.105 PMC5223290

[8] Rohban MH, Singh S, Wu X, Berthet JB, Bray M-A, Shrestha Y, et al Systematic morphological profiling of human gene and allele function via Cell Painting. Elife. 2017;6 doi: 10.7554/eLife.24060 28315521 10.7554/eLife.24060 PMC5386591

[9] Caicedo JC, Singh S, Carpenter AE. Applications in image-based profiling of perturbations. Curr Opin Biotechnol. 2016;39: 134–142. doi: 10.1016/j.copbio.2016.04.003 27089218 10.1016/j.copbio.2016.04.003

[10] Long F, Zhou J, Peng H. Visualization and Analysis of 3D Microscopic Images. PLoS Comput Biol. Public Library of Science; 2012;8: e1002519 doi: 10.1371/journal.pcbi.1002519 22719236 10.1371/journal.pcbi.1002519 PMC3375219

[11] Svoboda D, Ulman V. MitoGen: A Framework for Generating 3D Synthetic Time-Lapse Sequences of Cell Populations in Fluorescence Microscopy. IEEE Trans Med Imaging. 2017;36: 310–321. doi: 10.1109/TMI.2016.2606545 27623575 10.1109/TMI.2016.2606545

[12] Svoboda D, Kozubek M, Stejskal S. Generation of digital phantoms of cell nuclei and simulation of image formation in 3D image cytometry. Cytometry A. 2009;75: 494–509. doi: 10.1002/cyto.a.20714 19291805 10.1002/cyto.a.20714

[13] Rand WM. Objective Criteria for the Evaluation of Clustering Methods. J Am Stat Assoc. Taylor & Francis; 1971;66: 846–850.

[14] Ulman V, Maška M, Magnusson KEG, Ronneberger O, Haubold C, Harder N, et al An objective comparison of cell-tracking algorithms. Nat Methods. 2017;14: 1141–1152. doi: 10.1038/nmeth.4473 29083403 10.1038/nmeth.4473 PMC5777536

[15] Ljosa V, Sokolnicki KL, Carpenter AE. Annotated high-throughput microscopy image sets for validation. Nat Methods. 2012;9: 637 doi: 10.1038/nmeth.2083 22743765 10.1038/nmeth.2083 PMC3627348

[16] LeCun Y, Bengio Y, Hinton G. Deep learning. Nature. 2015;521: 436–444. doi: 10.1038/nature14539 26017442 10.1038/nature14539

[17] Krizhevsky A, Sutskever I, Hinton GE. ImageNet Classification with Deep Convolutional Neural Networks In: Pereira F, Burges CJC, Bottou L, Weinberger KQ, editors. Advances in Neural Information Processing Systems 25. Curran Associates, Inc; 2012 pp. 1097–1105.

[18] Ronneberger O, Fischer P, Brox T. U-Net: Convolutional Networks for Biomedical Image Segmentation. Medical Image Computing and Computer-Assisted Intervention–MICCAI 2015. Springer International Publishing; 2015 pp. 234–241.

[19] Abadi M, Barham P, Chen J, Chen Z, Davis A, Dean J, et al TensorFlow: A System for Large-Scale Machine Learning. OSDI. usenix.org; 2016 pp. 265–283.

[20] Jia Y, Shelhamer E, Donahue J, Karayev S, Long J, Girshick R, et al. Caffe: Convolutional Architecture for Fast Feature Embedding. Proceedings of the 22Nd ACM International Conference on Multimedia. New York, NY, USA: ACM; 2014. pp. 675–678.

[21] Yang SJ, Berndl M, Michael Ando D, Barch M, Narayanaswamy A, Christiansen E, et al Assessing microscope image focus quality with deep learning. BMC Bioinformatics. 2018;19: 28962. 10.1186/s12859-018-2087-4 PMC5853029 29540156

[22] Sadanandan SK, Ranefall P, Le Guyader S, Wählby C. Automated Training of Deep Convolutional Neural Networks for Cell Segmentation. Sci Rep. 2017;7: 7860 doi: 10.1038/s41598-017-07599-6 28798336 10.1038/s41598-017-07599-6 PMC5552800

[23] Sakurai Y, Kolokoltsov AA, Chen C-C, Tidwell MW, Bauta WE, Klugbauer N, et al Ebola virus. Two-pore channels control Ebola virus host cell entry and are drug targets for disease treatment. Science. 2015;347: 995–998. doi: 10.1126/science.1258758 25722412 10.1126/science.1258758 PMC4550587

[24] Stanley SA, Barczak AK, Silvis MR, Luo SS, Sogi K, Vokes M, et al Identification of host-targeted small molecules that restrict intracellular Mycobacterium tuberculosis growth. PLoS Pathog. 2014;10: e1003946 doi: 10.1371/journal.ppat.1003946 24586159 10.1371/journal.ppat.1003946 PMC3930586

[25] Wen Q, Goldenson B, Silver SJ, Schenone M, Dancik V, Huang Z, et al Identification of regulators of polyploidization presents therapeutic targets for treatment of AMKL. Cell. 2012;150: 575–589. doi: 10.1016/j.cell.2012.06.032 22863010 10.1016/j.cell.2012.06.032 PMC3613864

[26] Hartwell KA, Miller PG, Mukherjee S, Kahn AR, Stewart AL, Logan DJ, et al Niche-based screening identifies small-molecule inhibitors of leukemia stem cells. Nat Chem Biol. 2013;9: 840–848. doi: 10.1038/nchembio.1367 24161946 10.1038/nchembio.1367 PMC4009363

[27] Gibson CC, Zhu W, Davis CT, Bowman-Kirigin JA, Chan AC, Ling J, et al Strategy for identifying repurposed drugs for the treatment of cerebral cavernous malformation. Circulation. 2015;131: 289–299. doi: 10.1161/CIRCULATIONAHA.114.010403 25486933 10.1161/CIRCULATIONAHA.114.010403 PMC4356181

[28] Snijder B, Vladimer GI, Krall N, Miura K, Schmolke A-S, Kornauth C, et al Image-based ex-vivo drug screening for patients with aggressive haematological malignancies: interim results from a single-arm, open-label, pilot study. Lancet Haematol. 2017;4: e595–e606. doi: 10.1016/S2352-3026(17)30208-9 29153976 10.1016/S2352-3026(17)30208-9 PMC5719985

[29] Pennisi E. IMAGING. “Cell painting” highlights responses to drugs and toxins. Science. 2016;352: 877–878. doi: 10.1126/science.352.6288.877 27199393 10.1126/science.352.6288.877

[30] Caicedo JC, Cooper S, Heigwer F, Warchal S, Qiu P, Molnar C, et al Data-analysis strategies for image-based cell profiling. Nat Methods. 2017;14: 849–863. doi: 10.1038/nmeth.4397 28858338 10.1038/nmeth.4397 PMC6871000

[31] Dao D, Fraser AN, Hung J, Ljosa V, Singh S, Carpenter AE. CellProfiler Analyst: interactive data exploration, analysis and classification of large biological image sets. Bioinformatics. 2016; doi: 10.1093/bioinformatics/btw390 27354701 10.1093/bioinformatics/btw390 PMC5048071

[32] Ching T, Himmelstein DS, Beaulieu-Jones BK, Kalinin AA, Do BT, Way GP, et al Opportunities And Obstacles For Deep Learning In Biology And Medicine [Internet]. bioRxiv. 2017 p. 142760 doi: 10.1101/142760 10.1098/rsif.2017.0387 PMC5938574 29618526

[33] Rivron NC, Frias-Aldeguer J, Vrij EJ, Boisset J-C, Korving J, Vivié J, et al Blastocyst-like structures generated solely from stem cells. Nature. 2018;557: 106–111. doi: 10.1038/s41586-018-0051-0 29720634 10.1038/s41586-018-0051-0

[34] Roberts B, Haupt A, Tucker A, Grancharova T, Arakaki J, Fuqua MA, et al Systematic gene tagging using CRISPR/Cas9 in human stem cells to illuminate cell organization. Mol Biol Cell. 2017;28: 2854–2874. doi: 10.1091/mbc.E17-03-0209 28814507 10.1091/mbc.E17-03-0209 PMC5638588

