# Supplementary figures and images for: CellProfiler 3.0: Next-generation image processing for biology
Source: PLoS Biol. 2018 Jul 3;16(7):e2005970. doi: 10.1371/journal.pbio.2005970 (PMC6029841; doi:10.1371/journal.pbio.2005970)

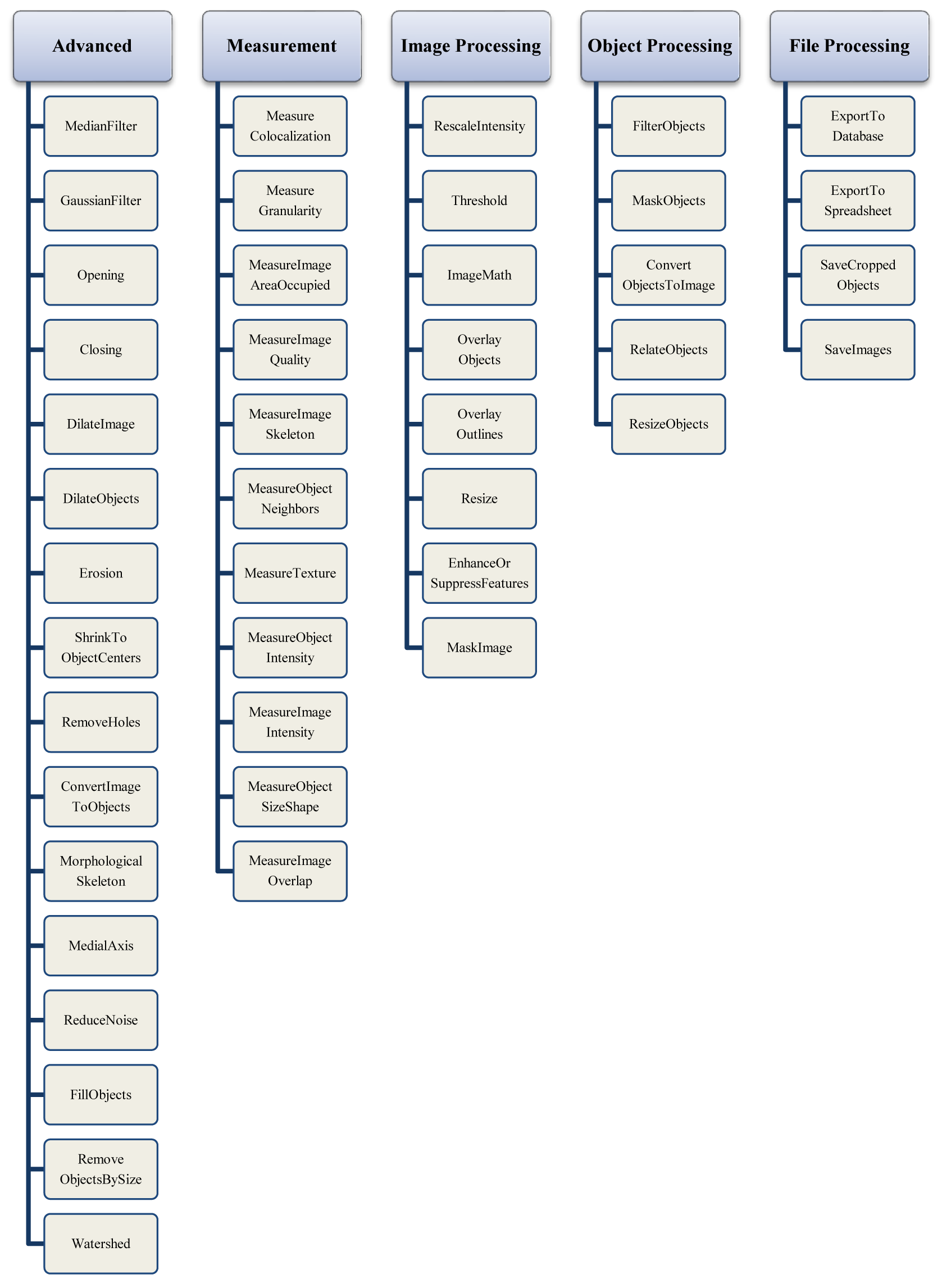

Supplement: S1 Fig — Overview of modules available in CellProfiler 3.1.0 for 3D image analysis. 3D, three-dimensional. (TIF) [file pbio.2005970.s001.tif]

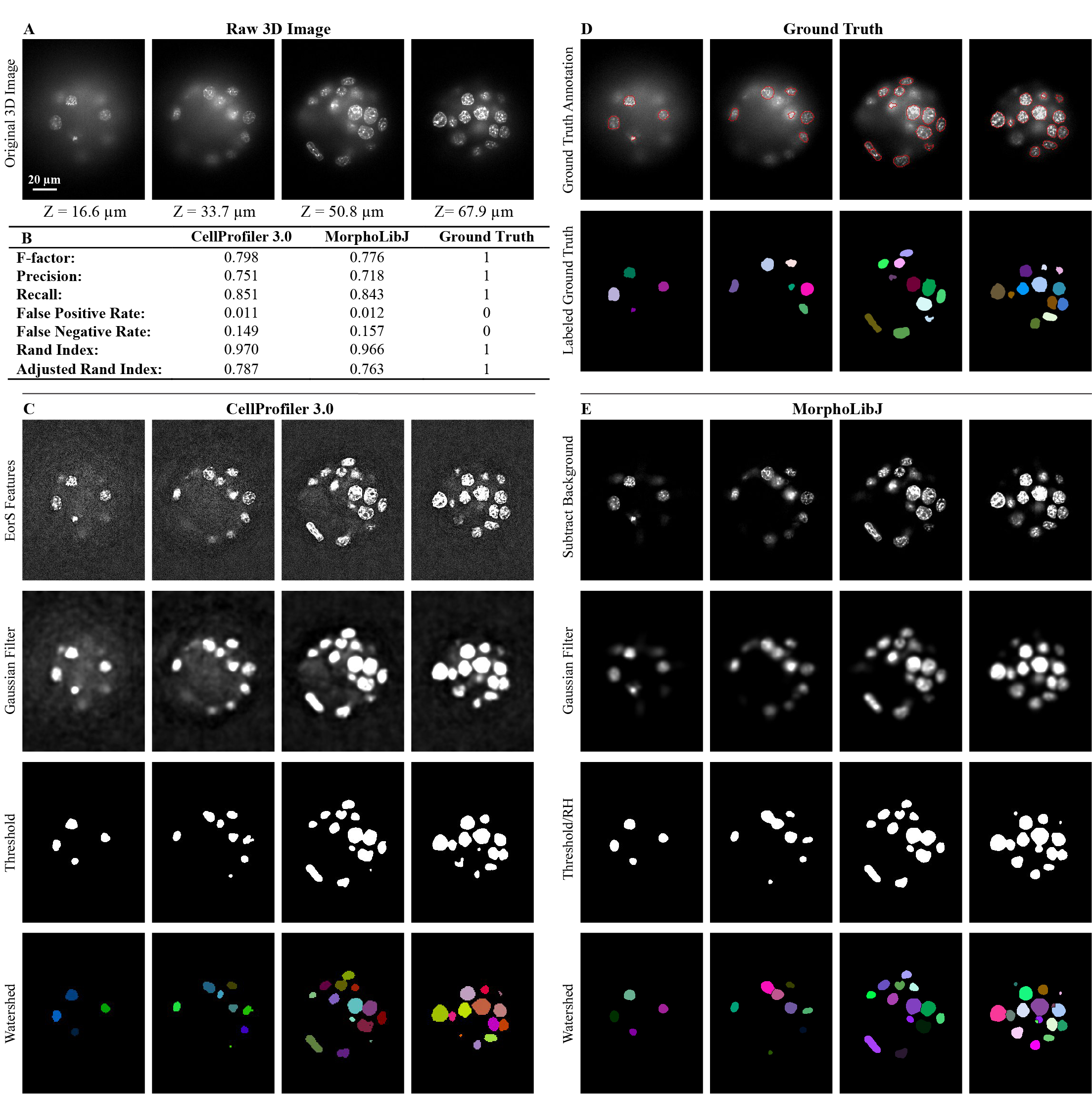

Supplement: S2 Fig — Images are available from the Broad Bioimage Benchmark Collection (https://data.broadinstitute.org/bbbc/BBBC032/), as in Fig 2A of the main paper. (A) Original 3D image of blastocyst nuclei prior to analysis. (B) Evaluation of CellProfiler 3.0 performance in comparison to the MorphoLibJ plugin in Fiji software. Both were compared to manually annotated ground truth using CellProfiler’s MeasureImageOverlap module. (C) CellProfiler 3.0 image processing modules used for blastocyst nuclei segmentation. (D) Ground truth obtained by manual annotation of each Z-slice using GIMP software. (E) Image processing done using Fiji’s MorphoLibJ plugin (macro code is presented in S1 Table). Images were obtained using PerkinElmer Ultraview VoX spinning disk microscope with a 63× immersion objective (distance between Z-slices = 0.5 μm) and provided by Javier Frias Aldeguer and Nicolas Rivron from Hubrecht Institute, Netherlands. 3D, three-dimensional. (JPG) [file pbio.2005970.s002.jpg]

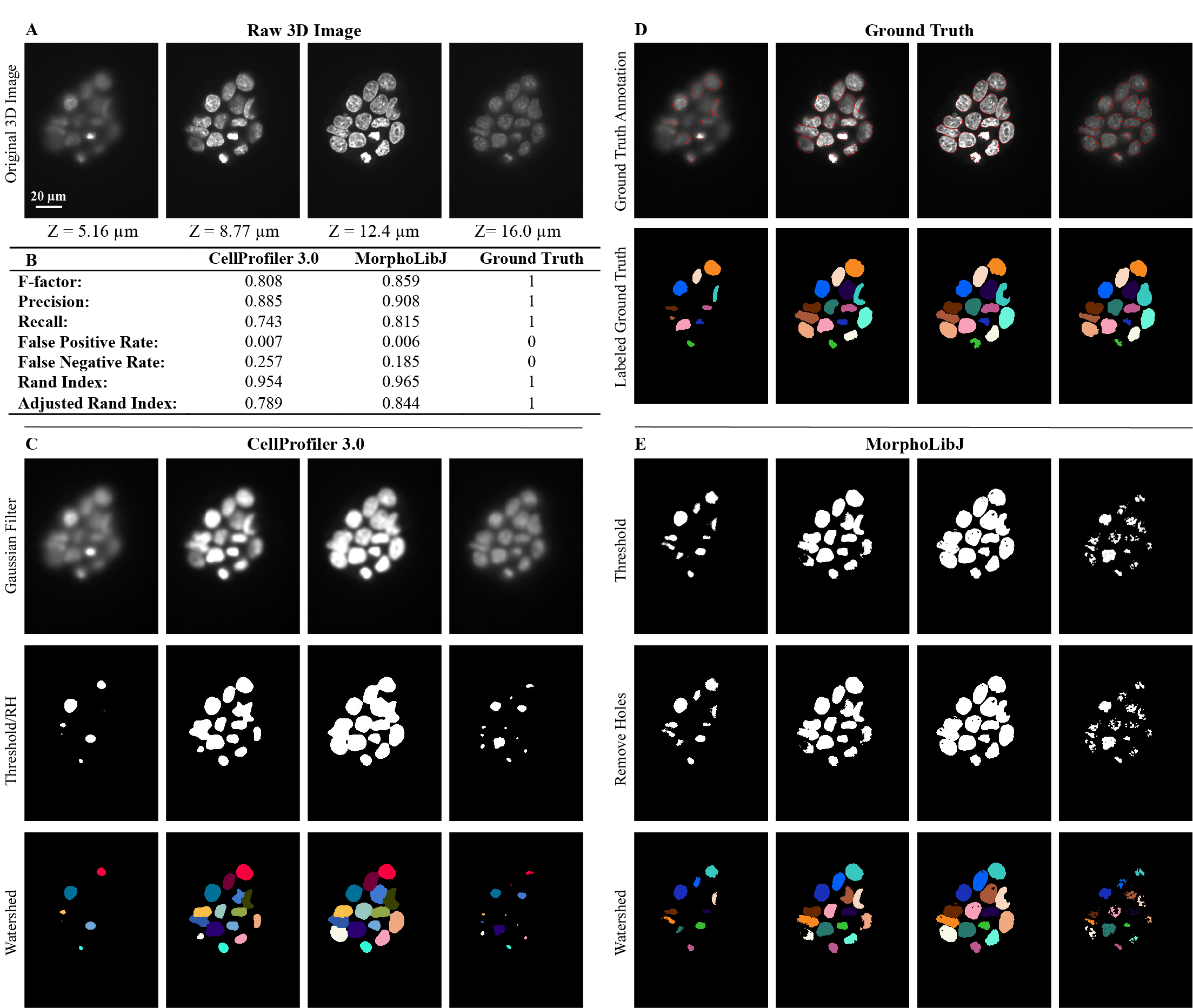

Supplement: S3 Fig — Images are available from the Broad Bioimage Benchmark Collection (https://data.broadinstitute.org/bbbc/BBBC033/), as in Fig 2B of the main paper. (A) Original 3D stem cell nuclei image prior to analysis. (B) Evaluation of CellProfiler 3.0 performance in comparison to the MorphoLibJ plugin in Fiji software. Both were compared to manually annotated ground truth using CellProfiler’s MeasureImageOverlap module. (C) CellProfiler 3.0 image processing modules used for stem cell nuclei. (D) Ground truth obtained by manual annotation of each Z-slice using GIMP software. (E) Image processing done using Fiji’s MorphoLibJ plugin (macro code is presented in S1 Table). Images were obtained using a PerkinElmer Ultraview VoX spinning disk microscope with a 63× oil immersion objective (distance between Z-slices = 0.5 μm) and provided by Javier Frias Aldeguer and Nicolas Rivron from Hubrecht Institute, Netherlands, and ground truth was annotated by Li Linfeng from MERLN Institute, Netherlands. 3D, three-dimensional. (JPG) [file pbio.2005970.s003.jpg]

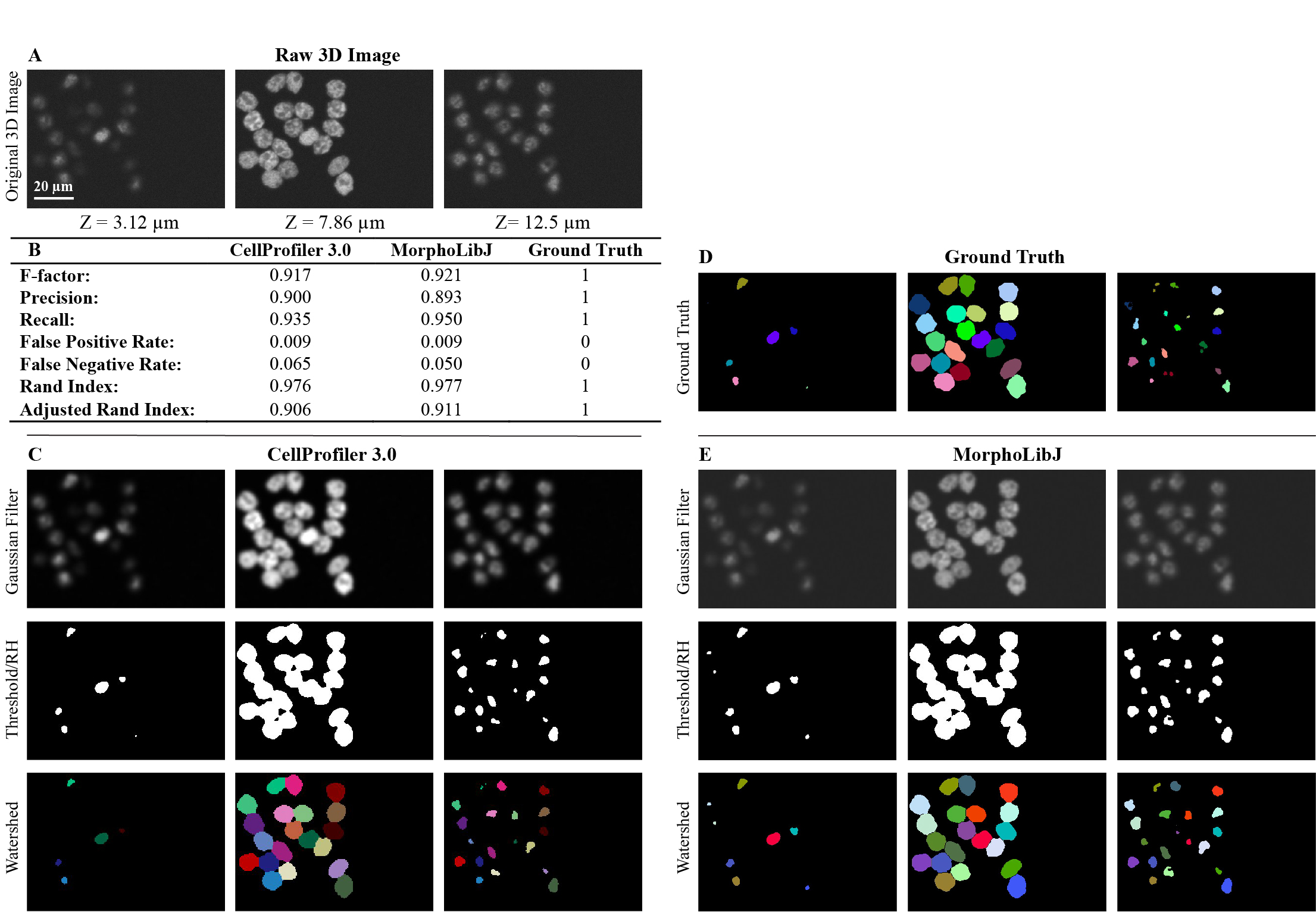

Supplement: S4 Fig — Images are available from the Broad Bioimage Benchmark Collection (https://data.broadinstitute.org/bbbc/BBBC024/), as in Fig 2C of the main paper. Synthetic images with 75% clustering probability and low SNR were chosen for analysis. The data set was generated using CytoPacq [12] set up to simulate a Zeiss Axiovert S100 microscope (objective Zeiss 63×/1.40 Oil DIC) attached to confocal unit Atto CARV and CCD camera Micromax 1300-YHS. (A) Original 3D image of HL60 nuclei prior to analysis. (B) Evaluation of CellProfiler 3.0 performance in comparison to the MorphoLibJ plugin in Fiji software. Both were compared to manually annotated ground truth using CellProfiler’s MeasureImageOverlap module. (C) CellProfiler 3.0 image processing modules used for HL60 cell nucleus segmentation. (D) Computer-generated ground truth. (E) Image processing done using Fiji’s MorphoLibJ plugin (macro code is presented in S1 Table). 3D, three-dimensional; CCD, charge-coupled device; SNR, signal-to-noise ratio. (JPG) [file pbio.2005970.s004.jpg]

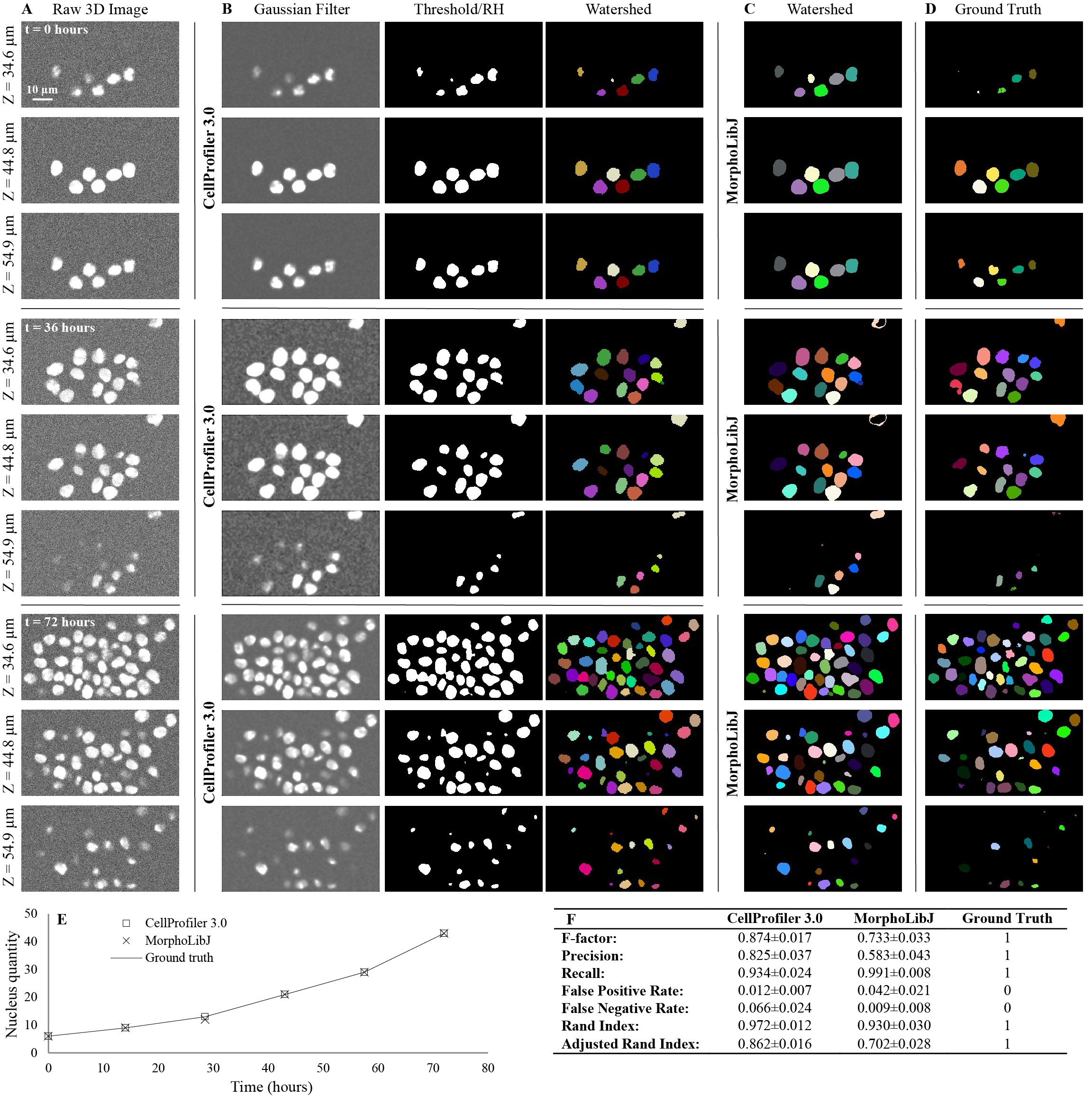

Supplement: S5 Fig — Images are taken from the Broad Bioimage Benchmark Collection (https://data.broadinstitute.org/bbbc/BBBC035/), as in Fig 2D of the main paper. (A) Original 3D image of HL60 nuclei prior to analysis. (B) CellProfiler 3.0 image processing modules used for HL60 cell nucleus segmentation. (C) Watershed obtained using Fiji’s MorphoLibJ plugin (macro code is presented in S1 Table). (D) Computer-generated ground truth. (E) Number of identified nuclei in six 3D images representing six different time points. (F) Evaluation of CellProfiler 3.0 performance (average and standard deviation of six images) in comparison to Fiji’s MorphoLibJ plugin. Both were compared to manually annotated ground truth using CellProfiler’s MeasureImageOverlap module. The data set was created by Vladimir Ulman and David Svoboda (Masaryk University, Czech Republic) using MitoGen, part of CytoPacq [12], to model a Zeiss Axiovert S100 microscope attached to confocal unit Atto CARV with a Micromax 1300-YHS camera with a Plan-Apochromat 40×/1.3 (oil) objective lens [11,14]. 3D, three-dimensional. (JPG) [file pbio.2005970.s005.jpg]

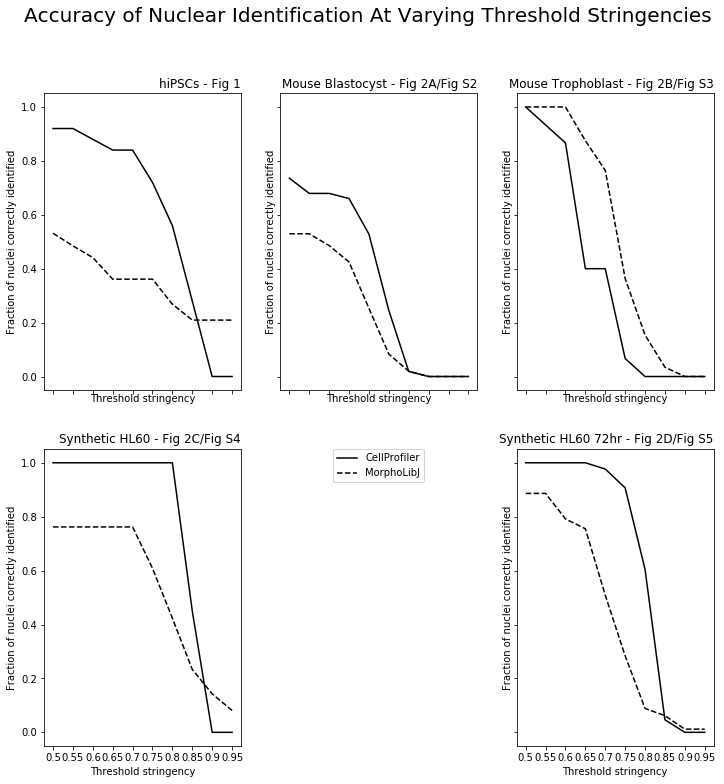

Supplement: S6 Fig — The fraction of nuclei correctly identified relative to their ground truth was assessed for both CellProfiler (solid line) and MorphoLibJ (dashed line) for the results shown in Fig 1 and S2–S5 Figs. A nucleus was considered correctly segmented at a given threshold if the intersection of the voxels of the ground truth and segmented nuclear volumes was greater than the threshold times the union of the voxels; small errors in segmentation are tolerated at lower thresholds but not at higher thresholds. CellProfiler met or exceeded the fraction correctly identified for most thresholds for 4 of 5 test images. Images and code needed to reproduce these results are available as S3 File. (PNG) [file pbio.2005970.s006.png]

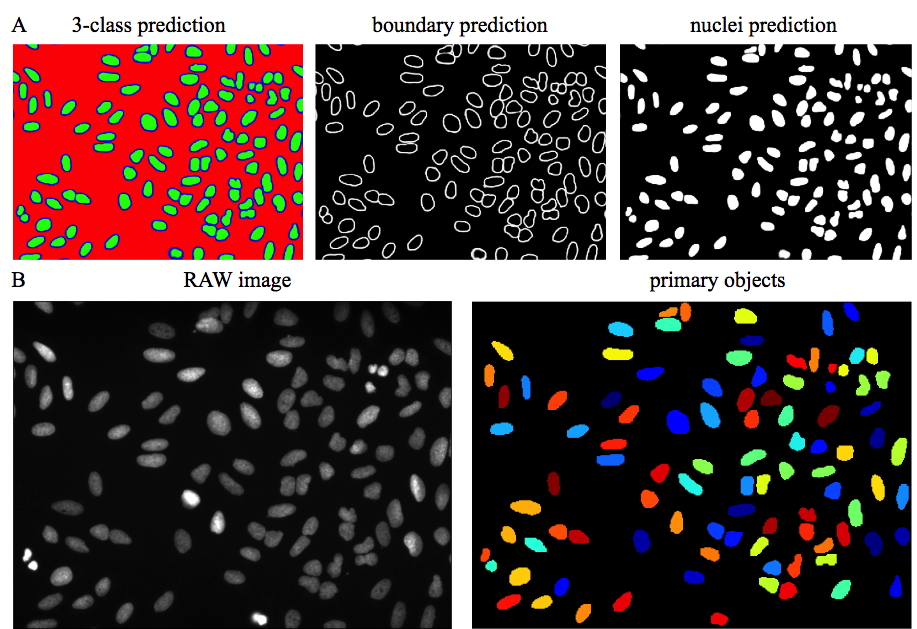

Supplement: S7 Fig — Image is available from the Broad Bioimage Benchmark Collection (https://data.broadinstitute.org/bbbc/BBBC022/, filename XMtest_B12_s2_w19F7E0279-D087-4B5E-9899-61971C29CB78.tif, see S4 File). The U-Net model was trained using 150 manually annotated DAPI images from the same collection. Implementation and training framework is available at https://github.com/carpenterlab/unet4nuclei. (A) The prediction for the three classes (background, boundary, and nuclei) is calculated for each image. (B) Raw image and nuclei segmentation using ClassifyPixels-Unet and IdentifyPrimaryObjects modules, with objects touching the edge excluded. Pipeline and image available as S4 File. (PNG) [file pbio.2005970.s007.png]

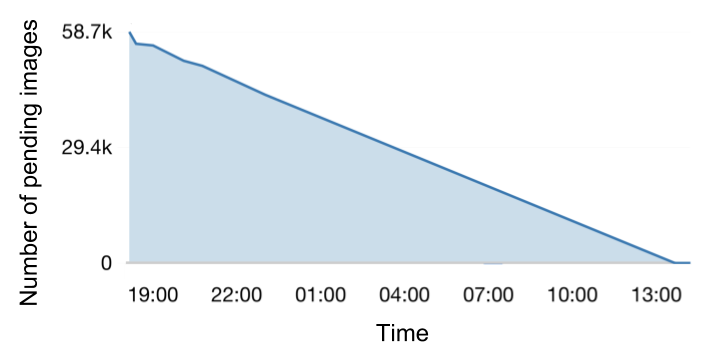

Supplement: S8 Fig — A data set of seventeen 384-well plates was processed using Distributed-CellProfiler on an AWS cluster. Each plate comprised 3,456 five-channel images (2,160 × 2,160 pixels). A CellProfiler pipeline was run on each image to identify cells and then extract measurements per cell. In all, 12,415,665 cells were identified, and 2,191 measurements were made per cell. It would have taken more than five months to analyze the data set using a single machine with 16 vCPUs. Using a cluster of 195 such machines on AWS, this data set was processed in less than 21 hours and cost $765 in total. The graph shows the number of pending images over the 21-hour period of processing this data set. The configuration files used to process this data set are provided in S5 File). AWS, Amazon Web Services; vCPU, virtual central processing unit. (PNG) [file pbio.2005970.s008.png]

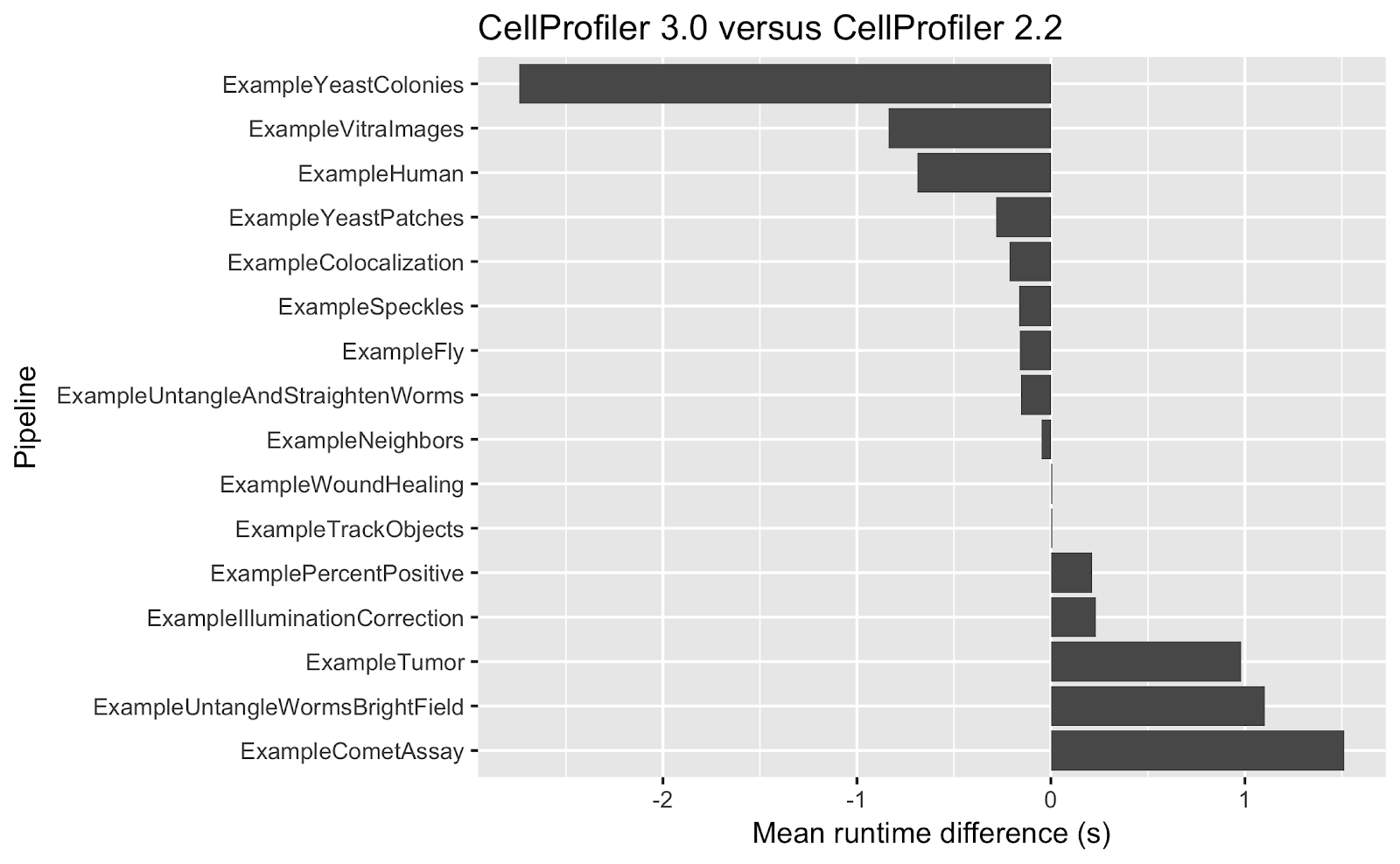

Supplement: S9 Fig — (Toward the left: CellProfiler 3.0 is faster; toward the right: CellProfiler 2.2 is faster). We compared the performance of example pipelines (available at https://github.com/CellProfiler/examples and http://cellprofiler.org) for CellProfiler 2.2 and 3.0 on OS X 10.12.6 (2.8 GHz Intel Core i7 and 16 GB 1600 MHz DDR3.). The figure above shows the difference of mean runtimes between CellProfiler 3.0 and 2.2 across 10 identical image sets. CellProfiler 3.0 demonstrates improved or comparable performance to CellProfiler 2.2 in 11 of 16 example pipelines, and these represent the more commonly used applications. (PNG) [file pbio.2005970.s009.png]

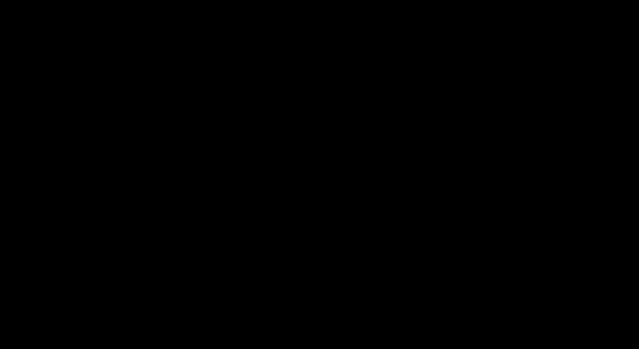

Supplement: S3 File — A file to reproduce the results presented in S6 Fig. This contains the ground truth images, CellProfiler-produced segmentations, and MorphoLibJ-produced segmentations, as well as a Jupyter notebook that can be run from the directory once unzipped to replicate the code. (ZIP) [file pbio.2005970.s014.zip › 5 - HL60 synthetic time/S5_ground_truth_segmented_149.tif]

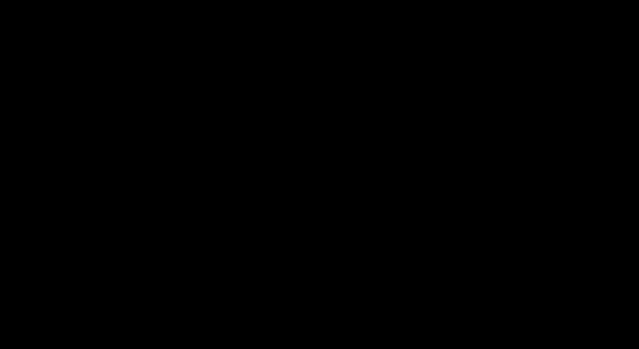

Supplement: S3 File — A file to reproduce the results presented in S6 Fig. This contains the ground truth images, CellProfiler-produced segmentations, and MorphoLibJ-produced segmentations, as well as a Jupyter notebook that can be run from the directory once unzipped to replicate the code. (ZIP) [file pbio.2005970.s014.zip › 5 - HL60 synthetic time/S5_t149_CellProfilerSegmentation.tiff]

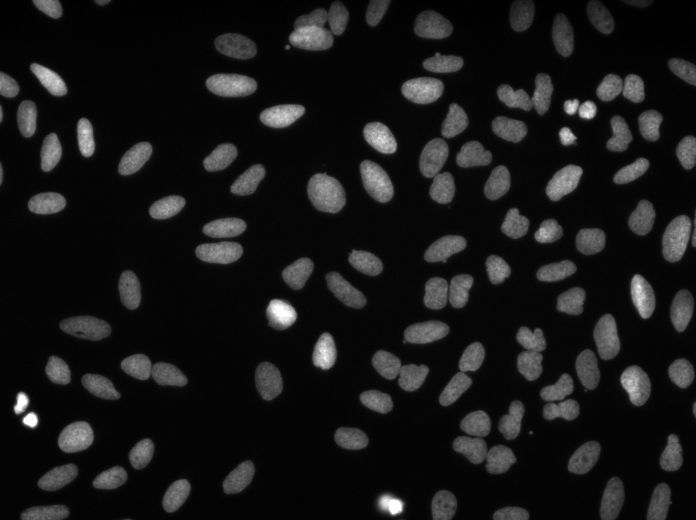

Supplement: S4 File — Example pipeline and image needed to run the ClassifyPixels-Unet module. (ZIP) [file pbio.2005970.s015.zip › IXMtest_B12_s2_w19F7E0279-D087-4B5E-9899-61971C29CB78.tif]
